# Determinants of perinatal mortality in Marondera district, Mashonaland East Province of Zimbabwe, 2009: a case control study

**DOI:** 10.4314/pamj.v8i1.71054

**Published:** 2011-02-02

**Authors:** Emmanuel Tachiweyika, Notion Gombe, Gerald Shambira, Addmore Chadambuka, Tshimamga Mufuta, Simukai Zizhou

**Affiliations:** 1 University of Zimbabwe, Department of Community Medicine PO Box A178 Avondale Harare, Zimbabwe; 2 Ministry of Health and Child Welfare, Zimbabwe

**Keywords:** Perinatal mortality, determinants, neonatal death

## Abstract

**Background:**

Marondera District recorded perinatal mortality ratios of 58.6/1000 and 64.6/1000 live births in 2007 and 2008 respectively. These ratios were above provincial averages of 32/1000 and 36/1000 during the same periods. We determined factors associated with perinatal mortality in Marondera District.

**Methods:**

A 1:2 unmatched case control study was carried out from June to August 2009. A case was any mother in Marondera District who had a stillbirth or early neonatal death from 01/08/2008 to 31/07/2009. A control was any mother whose baby survived the perinatal period during the same period. We calculated Odds Ratios and their 95% confidence intervals.

**Results:**

We interviewed 103 cases and 206 controls. Primary or no maternal education [OR=5.50 (3.14-9.33)] labor complications [OR=7.56 (4.38-13.06)], home delivery [OR=7.38 (4.03-13.68)] and preterm delivery [OR=15.06 (8.24-27.54)] increased the risk for perinatal mortality. Antenatal care booking [OR=0.19 (0.10-0.34)], having a gainfully employed husband [OR=0.36 (0.20-0.63)] and living within 5km of a health facility [OR=0.41 (0.22-0.78)] reduced the risk. Independent determinants of perinatal mortality included being apostolic [AOR=3.11 (1.05-9.18)], having a home delivery [AOR 7.17 (2.48-20.73)], experiencing labor complications [AOR=8.99 (3.11-25.98)], maternal HIV infection [AOR=5.36 (2.02-14.26)], antenatal care booking [AOR=0.32 (0.18-0.87)] and birth weight below 2500g [AOR=9.46 (3.91-27.65)].

**Conclusion:**

Labor complications, belonging to apostolic sect, having a home delivery, maternal HIV infection, low birth weight and antenatal care booking were independently associated with perinatal mortality. Health worker training in emergency management of obstetric and neonatal care was initiated. Marondera District started holding perinatal mortality meetings.

## Background

Globally, 3 million babies die in the first seven days of life (early neonatal period). It is estimated that more than 3.3 million babies are stillborn every year; one in three of these deaths occurs during delivery and could largely be prevented. In the less developed countries, which account for 98% of perinatal deaths, these deaths are not always registered [1]. World Health Organization (WHO) defines perinatal mortality as deaths occurring during late pregnancy (> 22 weeks of gestation), during birth and within seven days after delivery [2]. The perinatal period is considered the most critical phase of life [3,4]. It reflects the general health and the various socio-biological features of mothers and babies. Perinatal mortality rate gives a good indication of the extent of pregnancy wastage and the quality and quantity of health care available to mother and the newborn [5].

It would be difficult to achieve Millennium Development goal of reducing child mortality by two thirds by 2015 without reducing perinatal deaths [6]. Perinatal mortality rates are highest in Africa where it is more than six times higher than in developed regions. Perinatal mortality rates of around 10 deaths per 1000 total births are recorded in developed regions compared to 50 per 1000 in developing regions and over 60 per 1000 in least developed countries [7]. Perinatal deaths result from complications of preterm birth, asphyxia or trauma during birth, infections, severe malformations and other causes. Maternal health is important for neonatal health, and maternal infections contribute to adverse pregnancy outcomes [8]. The benefits of treating medical problems and complications of pregnancy are greatest when there is a continuum of care throughout pregnancy, child birth and immediate postpartum period. Although care during childbirth is most critical, antenatal care plays an important role, primarily because it provides an important means of addressing other health care needs, such as prevention and treatment of HIV, other sexually transmitted infections and malaria [9]. While international attention, statistics and interventions focus on infants born alive, stillborn infants have largely been overlooked [3]. A conceptual framework developed by Mosley was used to guide the study. It consists of distant factors (socio-economic factors, and pre-pregnancy conditions), pregnancy specific factors (nutritional status, maternal morbidity, intrapartum conditions, and antenatal care), and fetal factors (fetal biological factors and accidents at delivery) interacting leading to a perinatal death [10].

In 2007, Marondera District recorded 190 perinatal deaths and 3 242 live births, translating to a perinatal mortality ratio of 58.6 per 1000 live births. In 2008, the district recorded 145 perinatal deaths and 2 243 live births, translating to a perinatal mortality ratio of 64.6 per 1000 live births. These figures were above the provincial perinatal mortality ratio of 32 per 1000 in 2007 and 36 per 1000 in 2008 and the national perinatal mortality ratio which was estimated to be 27 per 1000 in 2008. These ratios might however be underestimates of the real problem given the gross underreporting of perinatal deaths and the high proportion of non-institutional deliveries in the district.

We determined factors associated with perinatal mortality focusing on socio-demographic factors, intrapartum factors, obstetric factors and health service factors.

## Methods

A 1:2 unmatched case control study was carried out in eleven health facilities in Marondera District in June and July 2009. A case was any mother who delivered a stillbirth or whose baby died within seven days of delivery in Marondera District from 01 August 2008 to 31 July 2009. A control was a mother whose baby survived the perinatal period in Marondera District during the same period. A minimum sample of 92 cases and 185 controls was calculated. We interviewed 103 cases and 206 controls.

Multistage stratified sampling was used to select health centers. The only district hospital and rural hospital were purposefully selected. One urban out of two urban clinics and eight out of 15 rural clinics were randomly selected using the lottery method. Maternity delivery registers were used to develop line lists of perinatal deaths which were used as sampling frames. Every second client on the perinatal death line lists was selected until the required number of cases was reached. Proportional sampling was used to determine the number of participants to be selected from each health center. Every second mother reporting for postnatal care was systematically selected as a control.

Interviewer administered questionnaires were used to collect data from study participants. Key informant interviews were done with provincial reproductive health managers, nurse managers and medical doctors. Discharged patients were followed up in their homes. A review of delivery registers, death notification forms and case notes was done. Epi-Info 3.3.2 was used to capture and analyze quantitative data. Stepwise logistic regression analysis was done to determine independent determinants of perinatal mortality. Qualitative data was analyzed for content.

## Results

**Demographic characteristics of study participants**

One hundred and three cases and two hundred and six controls were interviewed. The median years for cases was 25 years (Q_1_=22, Q_3_=30) and controls was 24 (Q_1_=20, Q_3_=28). table 1  shows the socio-demographic information of the cases and controls.

**Outcome of index pregnancies amongst cases**

Forty five (43.7%) of deliveries were live births and the remainder were stillbirths. Twenty four (41.4%) of the stillbirths were macerated and 34 (58.6%) were fresh.

**Figure 1:**
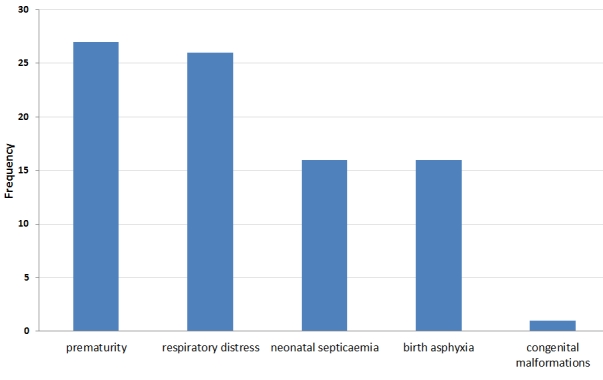
Causes of Early Neonatal Deaths among the Cases in Marondera District, Zimbabwe 2008-2009

Among the early neonatal deaths, 24 (53.3%) died in the first 24 hours of delivery, 18 (40%) died between 24 and 72 hours and 3 (6.7%) died between 72 hours and seven days. Of the 45 early neonatal deaths recorded, 27 died of severe prematurity, 26 of respiratory distress syndrome, 16 of neonatal septicaemia, 16 of birth asphyxia and 2 of congenital malformations. Some cases had more than one cause of death (Figure 1).

**Timing of antenatal care booking**

Fifty nine (57.2%) of cases and 181 (87.8%) controls had booked for antenatal care (p=0.000). Six (10.1%) cases and 35 (19.3%) controls booked in the first sixteen weeks of pregnancy. Forty (67.7%) cases and 102 (56.3%) controls booked between 16 and 27 weeks of gestation. Thirteen (22.2%) cases and 45 (24.4%) controls booked after twenty seven weeks of gestation (p=0.000).

**Use of partographs during labor**

Thirty-eight (36.8%) cases and 84 (40.7%) controls had their labor monitored using partographs.

**Determinants of perinatal mortality in bivariate analysis**

We carried out bivariate analysis to determine factors associated with perinatal mortality.

Socio-demographic factors associated with increased the risk of perinatal mortality were having maternal primary or no education (OR= 5.40, 95 % CI (3.14 – 9.33)), being in a polygamous marriage (OR=5.40, CI (1.62 – 11.26)), maternal parity greater than 4 (OR=4.80, CI (1.62 – 14.23)), living on a farm or in rural areas (OR=3.32, CI (1.93 – 5.74)), belonging to apostolic sect (OR=2.94, CI (1.70 – 5.08)) and maternal unemployment (OR=2.17, CI (1.24 – 3.75)). Living within 5 km of a health facility (OR=0.41, CI (0.22 – 0.77)) and having a gainfully employed husband (OR=0.36, CI (0.20 – 0.63)) reduced the risk of perinatal mortality.

Maternal determinants increasing the risk of perinatal mortality were having a history of early neonatal death (OR=7.22, CI (2.98 – 20.77)), having suffered from pregnancy complications (OR=7.04, CI (4.05 – 12.23)), having suffered from ante-partum haemorrhage (OR=5.60, CI (2.21 – 14.57)), maternal HIV infection (OR=5.30, (CI 2.68 – 10.56)), maternal history of pregnancy induced hypertension (OR=4.81, CI (2.59 – 8.99)), maternal history of stillbirth (OR=4.34, CI (2.26 – 8.37)), maternal history of abortion (OR=3.36, CI (1.90 – 5.96)) and having suffered from malaria during pregnancy (OR=4.32, CI (2.17 – 9.50)). Having booked for antenatal care (OR=0.19, CI (0.10 – 0.34)) reduced the risk of perinatal mortality.

Gestational age < 36 weeks (OR=15.1, CI (8.24 – 27.54)), home delivery (OR=7.38, CI (4.03 – 13.68)), birth weight  <2500g (OR= 6.26 (3.55 – 11.03)), baby having congenital malformation(s) (OR=5.62, CI (2.10 – 15.57)), and having multiple pregnancy (OR=3.26, CI (1.36 – 7.88)) were fetal determinants that increased the risk of perinatal mortality.

Labor related determinants of perinatal mortality were experiencing labor complications (OR=7.56, CI (4.38 – 13.06)), delivery by breech extraction (OR=3.59, CI (1.89 – 6.84)) and use of anesthesia during labor (OR=5.70, CI (1.34 – 27.77)), aggressive resuscitation of baby (OR=1.91, (CI 0.90 – 4.81)) and delivery by caesarian section (OR=2.39, CI (0.76 – 7.59)) increased the risk of perinatal mortality although they were not statistically significant. Having a spontaneous labor (OR=0.16, CI (0.02 – 0.89)) reduced the risk of perinatal mortality.

**Stratified analysis of determinants of perinatal mortality**

The association between birth weight and perinatal mortality was modified by gestational age. Preterm babies whose birth was < 2500g were more likely to die (OR= 11.43, CI (3.02 – 43.26)) than term babies (OR= 1.45, CI (0.62 – 3.59)) of low birth weight. Delivering at home was a confounder in the association between birth weight and perinatal mortality. Home delivery was causing an overestimate of the strength of association. Parity was a confounder in the association between pregnancy complications and perinatal mortality resulting in an overestimate of the strength of association.

**Independent risk factors for perinatal mortality**

Factors that were independently associated with perinatal mortality were labor complications, belonging to apostolic sect, having a home delivery, maternal HIV infection, low birth weight and antenatal care booking (table 2 ).

**Key informant interviews**

Key informants comprised of five nurse managers, three medical doctors, and one reproductive health coordinator. Their median years in service was 13 years (Q_1_=4, Q_3_=19). It was evident from the interviews that perinatal mortality meetings were not held according to schedule because of lack of funding. Key issues discussed during meetings included patient care, teaching of fellow health workers and perinatal mortality surveillance. Practical interventions for reducing avoidable deaths were discussed. Major causes of perinatal mortality highlighted include prematurity, HIV infection, birth asphyxia, pregnancy induced hypertension and home deliveries. Shortages of human and material resources were contributed to perinatal mortality.

## Discussion

The independent determinants of perinatal mortality were belonging to apostolic sect, antenatal care booking, home deliveries, experiencing labor complications, maternal HIV infection and low birth weight. Some apostolic sects do not use the formal health care system for obstetric care. Women from these apostolic sects deliver at home under the care of untrained midwives.

This invariably places the women and their babies at risk of complications and death. One of the pillars of the ‘Safe motherhood initiative’  is to have a clean delivery in a health institution under the care of trained health personnel [11]. Women who had booked for antenatal care (ANC) were less likely to experience perinatal mortality than those who had not booked. Antenatal care affords pregnant women the opportunity to have their pregnancies monitored and potential complications addressed. Women who delivered at home were more likely to experience perinatal mortality than those who delivered in health institutions. Home deliveries were often conducted by untrained birth attendants and in unsanitary conditions. Asphyxiated babies would not be resuscitated because of absence of equipment. There is an increased risk of neonatal infections and hypothermia in home delivered babies especially preterm babies. These factors are known risk factors for perinatal mortality [12].

Women who experienced labor complications had higher risk of perinatal mortality. Several studies have shown that complications (cord prolapse, mal-presentation, APH and eclampsia) to be associated with perinatal mortality. Babies delivered by breech extraction were often traumatized and asphyxiated during delivery. Babies delivered by caesarian section could die from the underlying complications that prompted emergency caesarian section rather than the procedure itself. Ferresu et al in 1998 however found that stillbirths were less likely to be delivered by caesarian section in a study to determine the incidence of perinatal deaths and their associated factors [13]. The risk of obstetric complications tends to increase with increasing parity thereby increasing the risk of perinatal mortality. Maternal parity greater than four was a risk factor for perinatal mortality. Pregnancy Induced Hypertension (PIH) and ante-partum hemorrhage (APH) increase the risk of perinatal mortality. APH is usually caused by placenta abruption and this result in fresh stillbirths. PIH reduces nutrient supply to the fetus due to restricted blood flow resulting in intrauterine growth retardation (IUGR). Such fetuses are too small and have high risk of dying in the perinatal period [14-15]. Similar findings were obtained in a study to determine the causes of perinatal mortality in WHO collaborating centers in Argentina, Egypt, Peru, India, South Africa and Vietnam in 2004 [16].

Low birth weight babies were more likely to die during perinatal period. Prematurity was the major cause of low birth weight. Preterm babies often died of hypothermia and respiratory distress syndrome. Premature low birth weight babies were however more likely to die during the perinatal period than low birth-weight term babies. Term babies would have better immunity and mature respiratory systems with adequate surfactant production and are able to regulate their body temperature [17]. Maternal infections increase the risk of perinatal mortality. Malaria causes placental insufficiency which leads to IUGR and sometimes intra-uterine death. Although Marondera District is considered a non-malaria area, several pregnant women referred from neighboring malaria infested districts would present with malaria at Marondera Hospital. Several studies have demonstrated that malaria is a risk factor for perinatal mortality [18].

Maternal HIV infection destroys the mother’s immune system and the infant depends on maternal antibodies to fight infection. The chances of survival are very low because of the compromised immunity passed on from the mother. Studies have shown that HIV infection increases perinatal mortality [19]. These observations can be related to social determinants of health. Women experiencing perinatal deaths are most likely to come from poor background. They may have to travel long distance on poor roads either on foot or rarely by vehicles for antenatal booking and care at a health care facility [20]. Where health facilities exist and are accessible, the quality of health care offered may be poor, due to understaffing or de-motivated health personnel.

Distance naturally prevents them from doing so even if they are knowledgeable of the benefits of ANC but deprives them the opportunity for early identification and management of pregnancy related problems and may further influence their choice of where to deliver. Because of poverty, women are less likely to afford a nutritious diet to take care of their needs and those of the growing foetus which in turn leads to low birth weight (LBW). Malnutrition increases risk of infection which leads to LBW babies with greater probability for mortality [21]. Residents living in regions with more poverty, more unemployment, and more income inequality are more likely to report poor health [13,22,23]. This is true of most rural areas in developing countries.

In India, the Government started a National Maternity Benefit Scheme (NMBS) program under which Rs. 500/-was given towards better food to every pregnant mother during the antenatal check-up as a measure to prevent malnutrition and another scheme that provided money for travel depending on distance from health facility. This increased interest in ANC, financial capability for ANC and timely transfer to referral facilities resulting in a decrease in perinatal mortality [24]. Maternal education is a factor that is considered valid for international comparisons. Studies from different European countries reported maternal education to be the most important social predictor of an adverse pregnancy outcome [25,26].

Similarly a review of studies from the Nordic countries showed educational level of the mother was the most important social factor associated with pregnancy outcomes [27] though in our study it was not independently associated. Perinatal mortality can be related to the paternal and maternal characteristics which may be related to gender power structures in families. But in programming very little focus is put on men’s role as partners in most aspects of reproductive health. Men play a critical role in contraception, abortion, control of sexually transmitted diseases, antenatal and delivery clinic attendance, and child mortality and should therefore be actively engaged to impact on women’s access to healthcare [28]. Most perinatal deaths were stillbirths and more than 58% of the stillbirths were fresh. A majority of these babies could have died during labor. Use of partographs for monitoring of labor was low and this could be attributed to shortage of midwives. More than 50% of early neonatal deaths occurred within 24 hours of delivery. Marondera Hospital had one incubator and sometimes up to three neonates would be nursed in one incubator. Simple interventions such as kangaroo care have the potential to reduce early neonatal deaths. The major causes of early neonatal deaths were prematurity, respiratory distress syndrome, neonatal septicemia and birth asphyxia.

**Study limitations**

Some records of labor were not complete and we could not get information concerning labor from the actual health workers who conducted the deliveries because of the retrospective nature of our study.

## Conclusion

The independent determinants of perinatal mortality in Marondera District include belonging to apostolic sect, experiencing labor complications, maternal HIV infection, home delivery, low birth weight and antenatal care booking. A majority of perinatal deaths were fresh stillbirths. Most early neonatal deaths occurred during the first 24 hours after delivery. Complications of prematurity and respiratory distress syndrome were the major causes of perinatal mortality in the district. Shortage of midwives and neonatal resuscitation equipment in most health facilities was contributing to poor outcomes in resuscitated babies. Transport and communication network in the district was poor. Perinatal mortality meetings were not being held according to schedule at all levels of health care. However in order to impact on perinatal mortality, it is important to look at the determinants in the context of health system and social circumstances that surround pregnancy and delivery.

We recommended that nurses should intensify health education to women on the importance of antenatal care booking and delivering in hospital. Hospital managers should ensure adequate provision of equipment and drugs for use in emergency obstetric and neonatal care. The District Nursing Officer should ensure that non-midwives in rural health centers are attached to the busy maternity unit at Marondera Hospital. The District Medical Officer (DMO) should urgently repair non-functional telephones and radios in peripheral health centers. Nurses should advocate for Kangaroo care method for preterm babies. Health workers should comprehensively manage neonates in the first 24 hours of delivery. Hospital and clinic managers should ensure that perinatal mortality meetings are held according to recommended schedule.

The Provincial Nursing Officer should consider re-opening of Marondera Hospital School of Midwifery so as to train more midwives. The reproductive health coordinator should ensure health worker training in Emergency Management of Obstetric and Neonatal Complications. The District Medical Officer (DMO) should increase the fleet of ambulances to efficiently service all health centers. The DMO should ensure adequate supply of neonatal resuscitation equipment and drugs in all health centers. The District Health Executive should consider construction of waiting mothers’ shelters because the district had none.

## Competing interests

The authors declare no competing interests. Source of funding: University of Zimbabwe, MPH Programme

## Acknowledgements

We would like to acknowledge the study participants for their valuable contributions that made this study a success.

## Authors’ contributions

Emmanuel Tachiwenyika: He was responsible for the conception of the problem, design, collection, analysis and interpretation of data and drafting the final article. Notion T. Gombe: He was responsible for the conception of the problem, design, analysis and interpretation of data and drafting the final article. Gerald Shambira: He was responsible for the conception of the problem, design, collection, analysis and interpretation of data and drafting the final article. Mufuta Tshimanga: Had oversight of all the stages of the research and critically reviewed the final draft for academic content. Addmore Chadambuka: Participated in the design, analysis and interpretation of data and drafting the final article and critical review of the final draft. Simukai T Zizhou: He was responsible for the design, analysis and interpretation of data and drafting the final article.

## Figures and Tables

**Table 1:** Socio-demographic information of cases and controls in Marondera District, Zimbabwe, 2009

**Variable**		**Cases**		**Controls**		**p-value**
		**n**	**%**	**n**	**%**	
Maternal age	<20 years	12	11.7	34	16.5	0.088
	20 – 35 years	82	79.6	165	80.1	
	> 35 years	9	8.7	7	3.4	
Median age		25 (Q_1_=22, Q_3_=30)		24 (Q1=20, Q3=28)		
Parity	0 – 4	92	89.3	201	97.5	0.002
	> 4	11	10.7	5	2.5	
Religion	Apostolic	45	43.7	43	20.9	0.000
	Orthodox	22	21.4	82	39.8	
	Pentecostal	21	20.4	57	27.7	
	Traditional	15	14.6	24	11.7	
Level of education	None	1	1.0	0	0	0.000
	Primary	63	61.2	48	23.3	
	Secondary	38	36.9	147	71.4	
	Tertiary	1	1.0	11	5.3	
Place of residence	Farm compound	44	42.7	57	27.7	0.000
	Growth point	2	1.9	0	0	
	Rural	31	30.1	35	17.0	
	Urban	26	25.2	114	35.3	
Marital status	Married	98	95.1	190	92.2	0.489
	Divorcee	3	2.3	5	2.4	
	Never Married	2	1.9	8	3.9	
	Widow	0	0	3	1.5	
Marriage type	Monogamous	67	68.4	175	92.1	0.000
	Polygamous	31	31.6	15	7.9	

**Table 2:** Independent determinants of perinatal mortality in Marondera District, Zimbabwe, 2009

Term	Adjusted Odds Ratio	95% CI	p-value
Belong to Apostolic sect	3.11	1.05 – 9.18	0.030
Home delivery	7.17	2.48 – 20.73	0.003
Booked for ANC	0.32	0.18 - 0.87	0.040
Labor complications	8.99	3.11 - 25.98	0.001
Mother HIV positive	5.36	2.02 – 14.26	0.008
Birth weight less than 2500g	9.46	3.91 – 27.65	0.000
